# Genomic Analysis Reveals Specific Patterns of Homozygosity and Heterozygosity in Inbred Pigs

**DOI:** 10.3390/ani9060314

**Published:** 2019-06-01

**Authors:** Ligang Wang, Yulian Mu, Linyang Xu, Kui Li, Jianlin Han, Tianwen Wu, Lan Liu, Qian Gao, Ying Xia, Guanyu Hou, Shulin Yang, Xiaohong He, George E. Liu, Shutang Feng

**Affiliations:** 1 Key Laboratory of Farm Animal Genetic Resources and Utilization of Ministry of Agriculture, Institute of Animal Sciences, Chinese Academy of Agricultural Sciences (CAAS), Beijing 100193, China; ligwang@126.com (L.W.); mouyulian@caas.cn (Y.M.); xulingyang@caas.cn (L.X.); likui@caas.cn (K.L.); h.jianlin@cgiar.org (J.H.); wtw198472@163.com (T.W.); liulan19789@163.com (L.L.); gaoqian36985@163.com (Q.G.); xiaying608@163.com (Y.X.); yangshulin@caas.cn (S.Y.); hexiaohong@caas.cn (X.H.); 2 Institute of Tropical Crop Variety Resources, Chinese Academy of Tropical Agricultural Sciences, Haikou, Hainan 571101, China; Guanyuhou@126.com; 3 Animal Genomics and Improvement Laboratory, U.S. Department of Agriculture-Agricultural Research Services, Beltsville, MD 20705, USA

**Keywords:** Wuzhishan pig (WZSP), genetic structure, inbred, homozygosity, heterozygosity

## Abstract

**Simple Summary:**

The inbred strain of miniature pig is an ideal model for biomedical research due to its high level of homozygosity. Our results from multidimensional scaling, admixture, and phylogenetic analyses indicated that the inbred Wuzhishan pig (WZSP), with its unique genetic properties, can be utilized as a novel genetic resource for pig genome studies. Inbreeding depression and run of homozygosity (ROH) analyses revealed an average of 61 and 12 ROH regions in the inbred and non-inbred genomes of WZSPs, respectively. ROH number, length, and distribution across generations indicate the impacts of recombination and demography on ROHs in these WZSPs. Finally, we detected 56 single nucleotide polymorphisms (SNPs) showing constant heterozygosity with heterozygosity (*He*) = 1 across six generations in inbred pigs, while only one was found in the non-inbred population. Among these SNPs, we observed nine SNPs located in swine RefSeq genes, which were found to be involved in signaling and immune processes. Together, our findings indicate that the inbred-specific pattern of homozygosity and heterozygosity in inbred pigs can offer valuable insights for elucidating the mechanisms of inbreeding in farm animals.

**Abstract:**

The inbred strain of miniature pig is an ideal model for biomedical research due to its high level of homozygosity. In this study, we investigated genetic diversity, relatedness, homozygosity, and heterozygosity using the Porcine SNP60K BeadChip in both inbred and non-inbred Wuzhishan pigs (WZSPs). Our results from multidimensional scaling, admixture, and phylogenetic analyses indicated that the inbred WZSP, with its unique genetic properties, can be utilized as a novel genetic resource for pig genome studies. Inbreeding depression and run of homozygosity (ROH) analyses revealed an average of 61 and 12 ROH regions in the inbred and non-inbred genomes of WZSPs, respectively. By investigating ROH number, length, and distribution across generations, we further briefly studied the impacts of recombination and demography on ROH in these WZSPs. Finally, we explored the SNPs with higher heterozygosity across generations and their potential functional implications in the inbred WZSP. We detected 56 SNPs showing constant heterozygosity with *He* = 1 across six generations in inbred pigs, while only one was found in the non-inbred population. Among these SNPs, we observed nine SNPs located in swine RefSeq genes, which were found to be involved in signaling and immune processes. Together, our findings indicate that the inbred-specific pattern of homozygosity and heterozygosity in inbred pigs can offer valuable insights for elucidating the mechanisms of inbreeding in farm animals.

## 1. Introduction

Inbred animals are an ideal model for conservation genetics, and can be utilized as a valuable genetic resource for human medical research [1]. Studying the genetic changes associated with inbreeding may provide novel insights for understanding genetic mechanisms and designing breeding strategies for specific phenotypes [2]. A run of homozygosity (ROH) is a genomic region of homozygous genotypes that are present in an individual due to parents transmitting identical haplotypes to their offspring [3]. ROHs have been previously explored in humans, cattle, pigs, and other farm animals [4,5,6,7,8]. The size and position of ROHs were expected to be associated with multiple causes, including recombination and demography [6,9]. Keller et al. found that inbreeding coefficients estimated from ROHs are much better at detecting the overall burden of rare recessive mutations (the likely cause of inbreeding depression) than several alternatives, including inbreeding coefficients defined based on single nucleotide polymorphism (SNP) and those defined from pedigrees [10].

Recently, high-throughput genotyping technologies have been successfully used to identify quantitative trait loci (QTL) that affect health, behavioral, meat quality, and production traits [11,12,13,14,15] and to explore population genetics in pigs [16,17,18,19,20,21,22]. Genome-wide genotyping data have significantly improved the well-established processes for pedigree testing and inbreeding estimation [22,23,24,25]. Previous studies also investigated the inbred Iberian pig’s functional genomics and population/conservation genetics using microsatellites, SNPs, and massive sequencing data [21,26,27,28,29,30,31,32].

The Wuzhishan pig (WZSP), characterized by its small adult body size, is a rare and endangered breed originating from the mountain area of Hainan province, China. From 1989, Shutang Feng and his colleagues have developed a highly inbred strain of WZSP based on the initial mating of one male with one female, followed by consanguineous mating for more than 24 generations [33,34]. A previous study also indicated no immune rejection occurred with the allograft among the inbred WZSPs [35]. With a high level of homozygosity and genetic stability, inbred WZSPs became an ideal animal model, as they have an almost identical genetic structure for laboratory experiments. For instance, previous studies have successfully used the inbred WZSP to investigate the structure of essential genes like swine leukocyte antigen-3 (*SLA-3*), nuclear element 1 (*PANE1*), and growth hormone receptor (*GHR*) to illustrate the mechanisms involved with immune response and dwarfism [36,37,38]. Recently, we conducted whole genome sequencing of the inbred WZSP, which demonstrated that over 60% of the genome exhibits extremely low rates of heterozygosis (<0.01%), indicating that inbreeding has eliminated a large proportion of heterozygosity from the genome [39]. However, heterozygosis remains in 30% of the genome of the sequenced individual. It remains largely unknown how ROHs change and their potential implications across generations during the inbreeding process.

In the current study, we performed genome-wide scan using the Porcine SNP60K Bead Chip in both inbred and non-inbred WZSPs. The non-inbred WZSP animals were sampled from a natural population, which was kept by random mating. After estimating their genetic structure, we determined and compared inbreeding coefficients based on the proportion of homozygotes in all pigs. We then investigated heterozygosity and ROH number, length, and distribution across generations. Finally, we explored their potential functional implications in the inbred WZSPs.

## 2. Methods and Materials

### 2.1. Ethics Statement

All animal procedures were performed according to guidelines developed by the China Council on Animal Care, and protocols were approved by the Animal Care and Use Committee of Chinese Academy of Agricultural Sciences, Beijing, China. The approval ID/permit numbers are SYXK (Beijing) 2008-007 and SYXK (Beijing) 2008-008.

### 2.2. Animals

Following the principles of inbred mice, an inbred WZSP strain was initiated from a pair of full-sibling WZSPs, an indigenous breed distributed in Hainan province, China. This strain was consanguineously bred and maintained up to the 24th generation by the Institute of Animal Science, Chinese Academy of Tropical Agricultural Sciences in Beijing. In this study a total of 96 inbred WZSPs, including 52 males and 44 females, were selected for analysis. They consisted of 16 animals for each of the six successive populations from the 17th (G17) to the 22nd (G22) generations. Another 12 animals of regular non-inbred WZSP maintained for conservation purposes in Hainan province were used as a control population. The population was re-created by mating several local types of mini-pigs in Hainan over the past two decades.

### 2.3. DNA Extraction and Genotyping

DNA was extracted from porcine blood leukocytes using the conventional phenol-chloroform protocol [40]. DNA concentration and quality were assessed using a NanoDrop ND-1000 spectrophotometer (NanoDrop Technologies, Wilmington, DE, USA). All genomic DNA samples were normalized to 50 ng/μL in a 96-well plate. Samples were genotyped with the Illumina’s Porcine SNP60K v2 BeadChip (Illumina, San Diego, CA) using the iScan platform (Illumina, San Diego, CA, USA) according to manufacturer’s instructions. Clustering and genotype calling were performed using the genotyping module implemented in the Genome Studio software (Illumina, San Diego, CA, USA). The mapping data was derived from Pig60K_SNP_pos_build10.2.txt.gz with 62,633 SNPs (last modified by Groenen et al. on 7 July 2014; http://www.animalgenome.org/repository/pig/).

### 2.4. Data Management and Quality Control

A total of 108 samples from inbred WZSPs and non-inbred pigs were genotyped using Illumina Porcine SNP60K. The samples showing a genotyping success rate of more than 98% were retained. A total of 52,258 raw SNPs were filtered with geno ≥ 0.01 (including only SNPs with a 99% genotyping rate or higher), and the Hardy–Weinberg equilibrium test (*p* <= 1 × 10^−5^). Finally, a total of 46,936 autosomal SNPs shared by the six generations of the inbred WZSP line and the non-inbred WZSP population were used for population genetic analysis. For file format conversion, data management, and quality control, we used PLINK v1.0.7 [41] software (http://zzz.bwh.harvard.edu/plink/) and custom-made in-house R scripts.

### 2.5. Genetic Diversity, Multidimensional Scaling and Admixture Analyses

Three metrics were used to estimate levels of breed genetic diversity: (1) Inbreeding coefficient based on the proportion of homozygotes, *F*; (2) Inbreeding coefficient based on ROH, *F*(ROH); and (3) heterozygosity (*H_e_*). The three metrics were estimated using PLINK v1.07 software. To investigate the genetic relationships between inbred strains and non-inbred population, multidimensional scaling (MDS) analysis was also conducted. To prevent uncorrected linkage disequilibrium (LD) from distorting the population stratification estimation, we pruned SNPs for LD using pair-wise genotype correction in 50 SNP sliding widows with a sliding step of 10 SNPs across the genome (indep-pairwise 50 10 0.2). The data pruning step resulted in 7714 SNPs for all 108 individuals.

Pairwise genetic distance (D) between individuals was calculated using PLINK, where D = 1 − (IBS2 + 0.5IBS1)/N). IBS1 and IBS2 are the number of SNPs that share either one or two alleles identical by state (IBS), respectively, and the N is the number of SNPs. Neighbor joining (NJ) trees were then built based on these two types of pairwise distances using PHYLIP 3.69 (http://www.phylip.com/). The phylogenetic trees were visualized in FigTree 1.3.1 (http://tree.bio.ed.ac.uk/software/figtree/). Clustering of breeds into genetic groups was examined using STRUCTURE 2.3.4 [42]. To avoid the bias that may be caused by LD and speed up the STRUCTURE runs, stringent LD filtering criteria were applied: date pruning with -indep-pairwise 50 10 0.02 resulted in 7714 SNPs for all 108 individuals in our analyses. STRUCTURE runs were performed using 10,000 replicates and 10,000 burn-in cycles under the admixture model and correlated allele frequencies.

### 2.6. Runs of Homozygosity Analysis and the Changes of Heterozygosity

To investigate homozygosity distribution in six inbred generations as well as the non-inbred population, we performed ROH analysis based on the 46,936 autosomal SNPs using PLINK v.1.07 with the following parameters: –homozyg-density 1000, –homozyg-window-het 1,and –homozyg-kb 10, –homozyg-window-snp 20 [6]. These ROH identification parameters included the required minimum density (1 SNP per 1000 kb), the number of heterozygous calls allowed within window 1, the threshold to identify an ROH (10 kb), and sliding window size (in 20 SNPs). The minimal size of an ROH was set to 10 kb. The region of ROH (ROHR) for each generation/group was defined as the nonredundant region merged across each generation/group. A comparison of the percentage and number of shared ROH regions for each inbred generation and the non-inbred population was conducted across individuals. We also briefly investigated the size of ROHs. ROHs were separated into three size classes: (1) small (<100 kb), (2) medium (0.1 to 5 Mb), and (3) large (>5 Mb) as described previously [6]. We computed the proportion of ROHs falling in each class across generation in inbred WZSP and non-inbred samples. Moreover, we estimated the heterozygosity for each generation and non-inbred pigs, and those SNPs with heterozygosity equal to one were used for gene enrichment analysis. Further analyses were carried out using the University of California Santa Cruz (UCSC) genome browser [43], to search for positional candidate genes located on the identified SNPs (positions were retrieved from the swine genome sequence assembly 10.2).

### 2.7. Gene Ontology and Pathway Analysis of Genes with He=1 SNPs

Gene ontology (GO) and Kyoto Encyclopedia of Genes and Genomes (KEGG) pathway analysis were carried out from the database for Annotation, Visualization and Integrated Discovery (DAVID, http://david.abcc.ncifcrf.gov) with reference genome v10.2. Fisher’s exact test was used to select each significant pathway, and the significance threshold was defined by the *p* value (*p* < 0.05).

## 3. Results

### 3.1. Estimation of Heterozygosity and Inbreeding Coefficient

**Figure 1:**
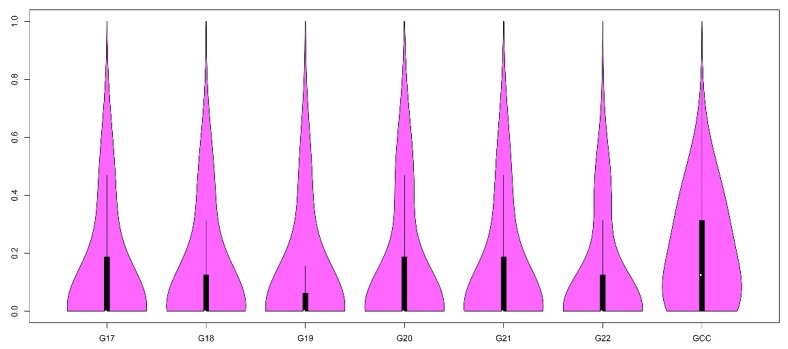
Violin plot showing the distribution of heterozygosity across generations G17 to G22 for inbred Wuzhishan pigs (WZSPs) as compared to non-inbred pigs. x-axis: different generations (G17–G22) in inbred WZSPs and non-inbred pigs (labelled as GCC); y-axis: the values of heterozygosity.

To determine the inbreeding-induced genetic changes in WZSPs over 24 generations of inbreeding, we conducted genetic diversity analysis of the non-inbred population and the inbred line from generations 17 to 22 using 52,258 autosomal SNP markers. We found that the average heterozygosity (*H_e_*) was clearly lower across six generations in the inbred WZSP compared to the non-inbred population (Supplementary Table S1). For instance, inbred strains from G17 and G22 displayed only ~50% heterozygosity (*He* ≈ 0.1) in the non-inbred population (*He* = 0.19). We observed 79 and 61 SNPs with heterozygosity greater than 0.5 (*He* > 0.5) and 0.8 (*He* > 0.8) in all six inbred generations (G17 to G22), respectively (Figure 1). Interestingly, we found average *He* showed a slight increase for G20 across time, and then displayed a decreasing trend in G22. Notably, we found some SNPs displayed extremely high heterozygosity with *He* = 1, including 56 SNPs in the inbred strain and five SNPs in the non-inbred population. Among the 56 SNPs, only one locus appeared in both groups. 

To estimate the degree of inbreeding in inbred WZSPs, we calculated the average inbreeding coefficient *F* and *F(ROH)* across generations (Supplementary Table S2A). High correlation was observed between the two measures of inbreeding (Supplementary Table S2B). Since *F(ROH)* is better at detecting both rare and common variants, we focused on *F(ROH)* for downstream analysis. We found the average *F(ROH)* value across inbred populations was 0.49. Among six generations, the highest average inbreeding coefficients *F(ROH)* was 0.52 in G22, while the relatively low *F(ROH)* was found in G20 and G21. We conducted Student’s *t*-test on *F(ROH)* values between generations and groups (Supplementary Table S2C). We found that most generations in inbred WZSP showed significant difference with *p*-value < 0.01 when compared with non-inbred pigs.

### 3.2. Genetic Structure Estimation for the Inbred WZSP

**Figure 2:**
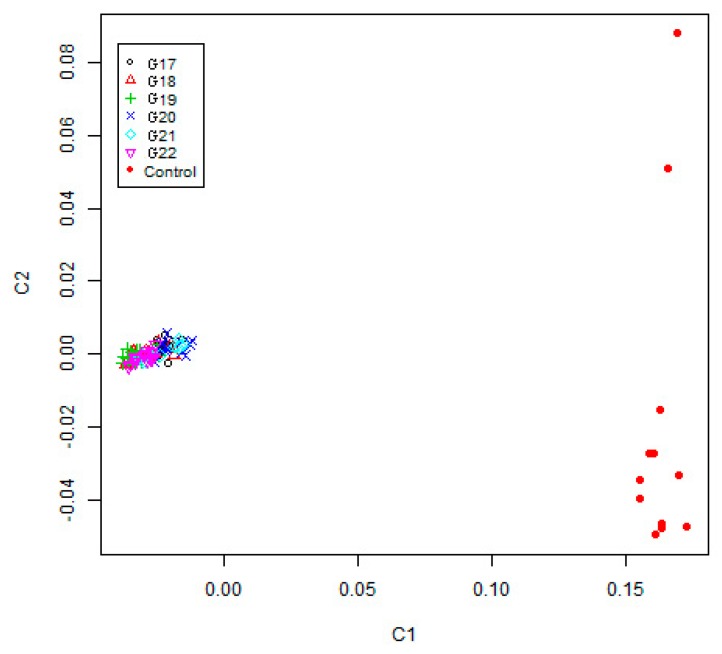
Multidimensional scaling analysis of the 108 individuals including 96 inbred strain and 12 non-inbred individuals. This analysis is based on genome-wide identity-by-state pairwise distances calculated with the PLINK software using 7714 linkage disequilibrium (LD)-filtered single nucleotide polymorphisms (SNPs). C1: first component; C2: second component.

To estimate the genetic difference between inbred and non-inbred pigs, we performed MDS analysis on 108 individuals (96 inbred and 12 non-inbred) using 7714 LD-filtered SNPs. In this study, we observed that the studied individuals displayed clear population stratification (inbred versus non-inbred) for the first component (C1) and the second component (C2) (Figure 2). This is consistent with the inbred selection history whereby inbred WZSPs were developed from one male and one female via full-sibling mating. Also, we found the non-inbred pigs were well separated and placed along the second gradient of genetic variations. Moreover, genetic structure for inbred individuals from G17 to G22 cannot be clearly distinguished using the first component (C1). Our analysis revealed that the individuals across generations G17~22 displayed similar genetic structures. In addition, we observed 96 inbred pigs were intermixed in our multidimensional scaling analysis, and cannot be separated by generations. Our results thus support the patterns of homozygosity obtained.

**Figure 3:**
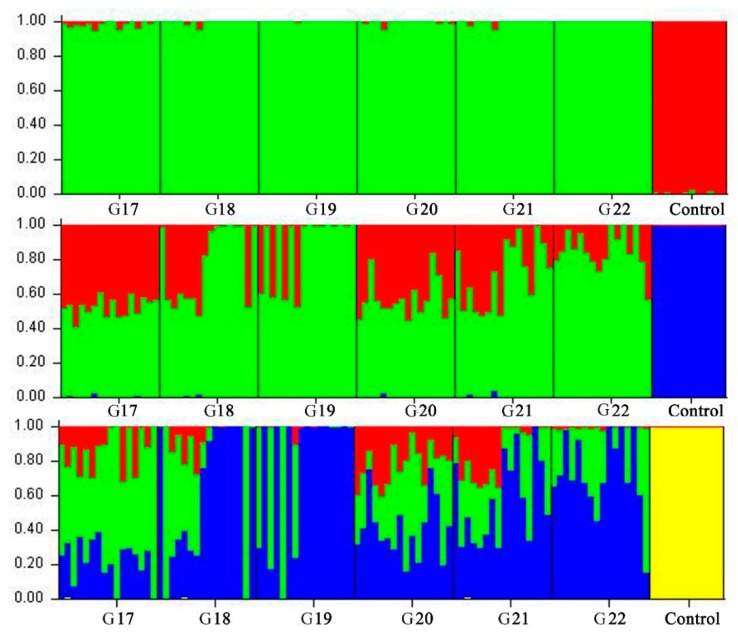
Population genetic structure and admixture estimation in 96 inbred WZSPs and the non-inbred population. From the top to the bottom is the clustering of 108 individuals when K = 2, 3, and 4, respectively. Individuals are shown as a thin vertical line colored in proportion to their estimated ancestry.

**Figure 4:**
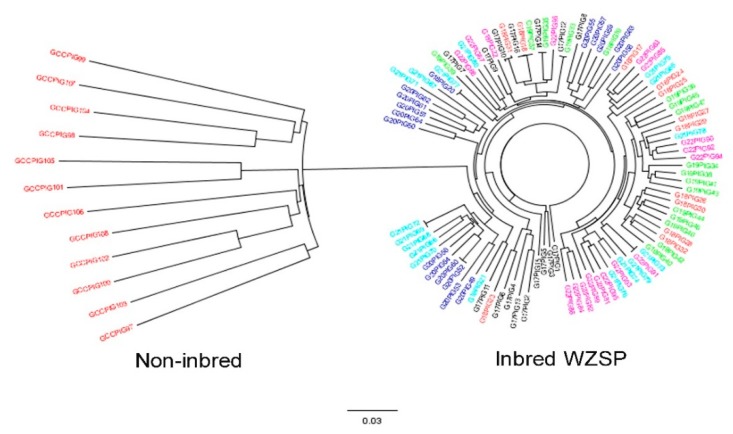
Neighbor-joining tree of the 96 inbred pigs and 12 non-inbred pigs. Individual and breed relationships among 108 pigs illustrated by genetic distances estimated using 7714 LD-filtered SNPs.

We further examined inbred and non-inbred WZSPs using admixture analysis in STRUCTURE 2.3.4 (Figure 3). We found the results at K = 2 were consistent with MDS analysis. When assuming K = 3 and 4, we found the inbred WZSP could not be separated by generation, indicating similar genetic structures among individuals across generations. We finally constructed NJtree based on genetic distances (Figure 4). Although inbred and non-inbred WZSP can be easily distinguished, we observed individuals from inbred WZSP in six generations were mixed together, and this result was also in agreement with MDS and admixture analyses. Taken together, our results provided strong evidence to support the conclusion that inbred WZSPs have higher levels of homozygosity than pigs in the non-inbred population.

### 3.3. Runs of Homozygosity and ROH Regions across Generations

We observed significant differences of ROH occurrence between the inbred WZSP and non-inbred population. On average, 61 ROHs per individual (1300 Mb per individual, occupying about 53% of genome) were detected in inbred WZSPs, whereas on average 12 ROHs per individual (173.8 Mb per individual, corresponding to 6.68% of genome) were identified in the non-inbred population. In inbred WZSPs, we found the largest ROH proportion in G22, covering 1830 Mb in the pig genome (~70% of genome). In contrast, we observed a relatively small proportion of ROHs (<0.5 Mb) in the non-inbred population. To further explore the characteristics of ROHs for inbred and non-inbred WZSPs, we merged ROHs detected from individuals into nonredundant regions (ROHR) for each generation. We observed the estimated ROHR for each generation in inbred WZSPs was dramatically larger than that of the non-inbred population. Then, we merged the ROHs from each individual to a subset of ROHR across 108 individuals, and found a total of 22 ROHR distributed at 18 autosomes, with some regions occupying nearly the whole chromosome. The two largest regions were found on chromosomes 1 and 17, whereas the smallest region was observed on chromosome 11 at 5.3 Mb. Among 22 ROHRs, we observed that the count of ROHs for each generation in inbred WZSPs was about 2~4 fold of that of non-inbred population. Moreover, we found 70, 74, 71, 68, 77, and 72 ROHs at chromosome 1 in generations 17–22, respectively, while only there were 45 ROHs in the non-inbred population. Also, for the ROHR14 at chromosome 11, we found 10, 6, 8, 2, 2, and 3 ROHs in generations 17–22, and two ROHs in the non-inbred population, respectively (Supplementary Table S3). The detailed summary statistics for ROHR are presented in the additional file (Supplementary Table S3).

### 3.4. ROH Number, Length, Distribution, and Their Implications

**Figure 5:**
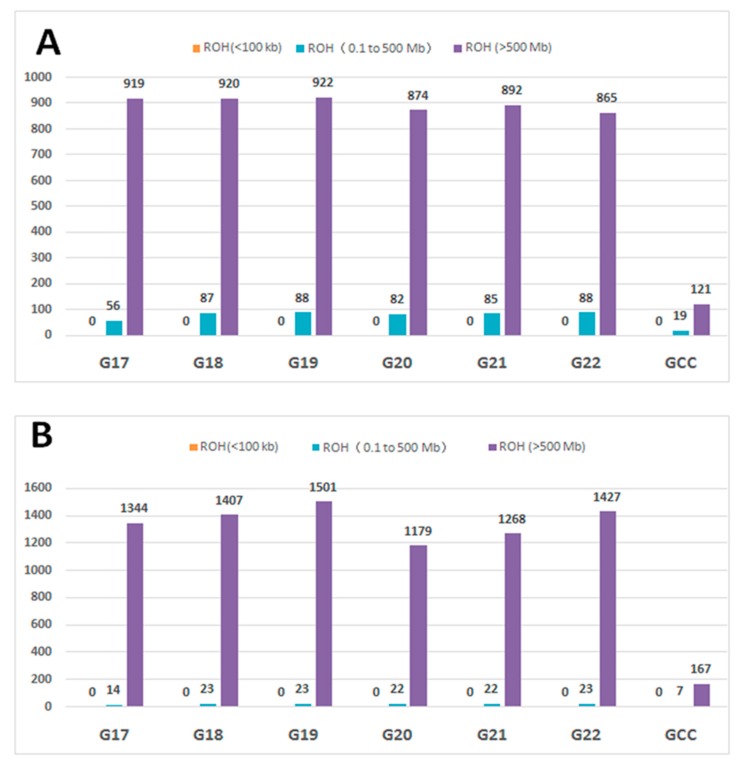
Total number of runs of homozygosity (ROHs) and average size of the genome covered by ROHs across inbred and non-inbred WZSPs. (**A**) The number of ROHs belonging to the three size classes: small (<100 Kb), medium (0.1 to 5 Mb), and large (>5 Mb) for each generation in inbred and non-inbred WZSPs. y-axis: the total number of ROHs. (**B**) The average size of the genome that is covered by the particular ROH size class in one individual averaged for each group. y-axis: the average ROH size in Mb.

**Figure 6:**
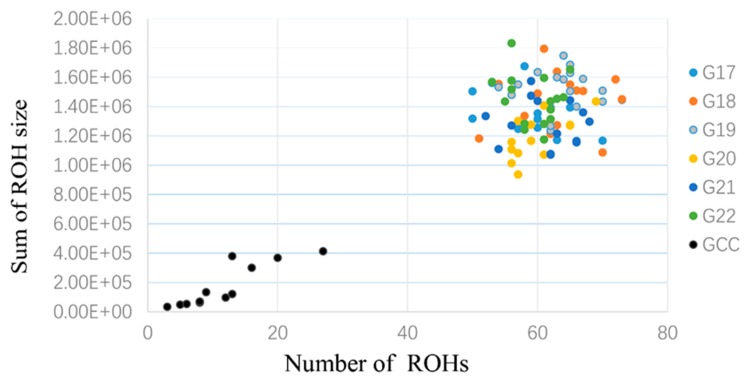
Number of ROHs and sum of ROH sizes for each inbred and non-inbred WZSP. Number of ROHs and sum of ROHs detected for each of 108 individuals genotyped by the Illumina Porcine 60 K SNP chip. Non-inbred pigs are shown in black, and six generations in inbred pigs are indicated by various colors.

**Figure 7:**
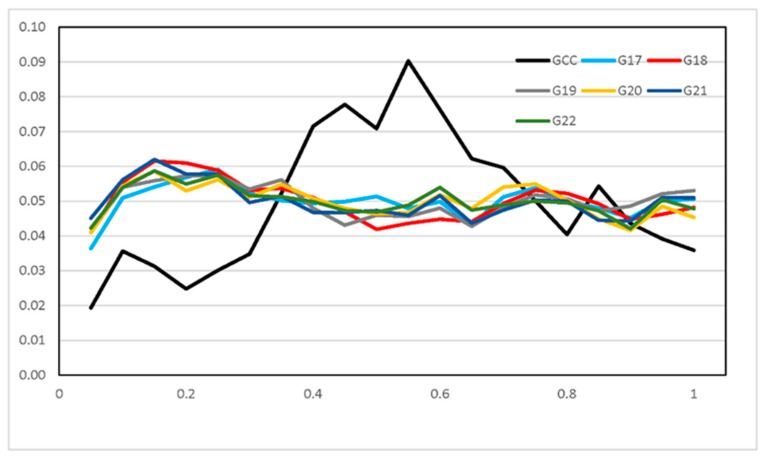
Distribution of ROHs for inbred and non-inbred WZSPs along a chromosome. The relative chromosomal position on a chromosome was calculated by standardizing the original physical position by the chromosome length: a value of zero corresponds to the beginning of a chromosome and a value of one corresponds to the end. The distributions are averaged across all chromosomes. Non-inbred pigs are shown in black, and six generations in inbred pigs are indicated by various colors. x-axis: the relative chromosomal position on a chromosome; y-axis: relative frequency of ROHs.

ROHs were further separated into three size classes: (1) small (<100 Kb), (2) medium (0.1 to 5 Mb), and (3) large (>5 Mb) as described previously [6]. We computed the proportion of ROHs falling in each class across generations in inbred WZSPs and non-inbred samples. No ROH was found for small size across populations (Figure 5). In contrast, large ROHs were more common and covered significantly more of the genome than medium ROHs. The large ROHs were at least 10-fold more abundant than medium ROHs. We also plotted number and cumulative ROH size for these 108 individuals from the inbred and non-inbred WZSPs (Figure 6). As we described before, in the non-inbred pigs the number of ROHs ranged from 3 to 27 and cumulative ROH size ranged from 34.6 Mb to 413.5 Mb. Inbred pigs had a much larger number of ROHs and cumulative ROH size, forming a distinct mixed cluster as compared to non-inbred pigs. As reported before, the likelihood of ROH occurrence at a particular chromosomal position was dependent on the size of the ROH [6]. We also checked their distributions along the chromosomes. For the non-inbred pigs, the large ROHs appeared more in the low recombination regions in the middle of the chromosome, and much less towards the telomeric regions (Figure 7). For inbred WZSPs, the ROH seemed to be evenly distributed across the chromosomes in all generations.

### 3.5. Functional Gene related to the Change of Heterozygosity

In this study, we found some SNPs displayed complete heterozygosity with *He* = 1, including 56 SNPs in inbred WZSP and five SNPs in non-inbred pigs. In total, we observed nine SNPs located in known RefSeq gene (Table 1). Among them, we found five genes, including estrogen receptor 1 (*ESR1*), receptor-interacting serine-threonine kinase 2 (*RIPK2*), FYVE, RhoGEF, and PH domain containing 2 (*FGD2*), ubiquitin specific peptidase 4 (proto-oncogene) (*USP4*), and Sus scrofa nuclear receptor corepressor 2 (*NCOR2*) which were involved in the pathway of G protein-coupled receptors; and three genes including ferredoxin 1 (*FDX1*), abhydrolase domain containing 5 (*ABHD5*), and 6-phosphofructo-2-kinase/fructose-2, 6-biphosphatase 4 (*PFKFB4*) which were involved in metabolism process. One gene, named protein kinase, cAMP-dependent regulatory type II alpha (*PRKAR2A*), was involved in platelet activation, signaling and aggregation, and sertoli–sertoli cell junction dynamics.

## 4. Discussion

Our previous study had revealed that genomic regions with extremely low rates of heterozygosis (<0.01%) account for 60% of the genome in one sequenced inbred WZSP [39]. In this study, we investigated genetic structure using multidimensional scaling, genetic admixture, and phylogenetic analyses for six generations of inbred and non-inbred WZSPs, and our results strongly suggested inbred that WZSPs are distinct from non-inbred pigs. We also conducted ROH analysis to investigate inbreeding level across six generations using the Porcine SNP60K array. We revealed that inbred WZSPs have been under strong inbreeding in the past three decades based on genetic diversity estimation. We observed a large number of ROHs were shared across inbred generations, which may imply most of these ROHs can be transmitted stably across generations. 

On the other hand, we also investigated heterozygosity persistence across generations in inbred WZSPs. Our results shed light on the functional aspects of genes which remained heterozygous across generations. These genes included *ESR1*, *RIPK2*, *FGD2*, *USP4*, *NCOR2*, *FDX1*, *ABHD5*, *PFKFB4*, and *PRKAR2A*, which are involved in signaling and other important processes. This was in accordance with general observations that genes involved in pathogen resistance appear to comprise a significant proportion of the polymorphisms known to be maintained by the heterozygote advantage [44,45]. The heterozygote advantage describes the situation where the heterozygote at a locus with two alleles has a higher fitness than both homozygotes. Heterozygote advantage is often used synonymously with overdominance to explain heterosis. Our earlier investigation on heterozygosis of the WZSP inbred diploids revealed an unexpectedly high rate of polymorphism maintained in certain genomic regions [39]. However, the mechanism to explain why high heterozygosis was maintained during inbreeding remains unclear. Recessive lethality may be one reason, but it cannot be the explanation for all heterozygous regions, which account for more than 30% of the genome. 

Since both recombination and selection have influence on pattern of ROHs, we studied ROH number, size, and distribution and provided some evidence for the correlation between ROH and recombination. The fact that cumulative ROH size was dominated by large ROHs in inbred and non-inbred WZSPs may be due to the limited numbers of small ROHs in WZSPs and/or due to the fact that small ROHs cannot be efficiently detected due to the limited resolution of the SNP chip. Even so, in non-inbred WZSPs we found that the large ROHs appeared more in the low recombination regions in the middle of the chromosome, and less towards the telomeric regions. The inbred WZSP, by contrast, showed a more uniform distribution of ROHs across chromosomal position. This may suggest that large ROHs were not yet broken down by recombination in inbred WZSPs due to their short history, while they were broken down by recombination at chromosomal terminals in a non-uniform distribution in non-inbred WZSPs. Therefore, it was revealed that ROHs might reflect both the recent past and current status of a population as well as distant population history, and are very susceptible to population dynamics.

As genetic drift may occur in a population of limited size during the inbreeding process, we cannot totally exclude this possibility but believe the probability is low. A further population study of WZSPs will enable us to distinguish whether heterozygous genes resulted from selection or recombination.

Sequencing of non-inbred WZSPs as an out-group and the inbreeding pedigree will be important for future high-resolution studies and should help us to understand how heterozygosis decreased or was maintained, and how chromosome structures reorganized during the inbreeding process from generation to generation.

## 5. Conclusions

In summary, to our knowledge this study is the first attempt to explore the genetic characteristics of an inbred pig strain at a genome-wide level. We found significant genetic differences between inbred and non-inbred pigs. Moreover, we revealed and confirmed that several SNPs maintained higher heterozygosis even in inbred WZSPs. Our results offer some valuable insights into understanding of the pattern of homozygosity and heterozygosis during the inbreeding process.

## Figures and Tables

**Table 1:** The nine identified SNPs with *He* = 1 and their related functional genes and Gene ontology (GO) terms and Kyoto Encyclopedia of Genes and Genomes (KEGG) pathways.

Chr	Pos ^1^	Gene	Gene Accession Number	Gene Involved GO Terms and KEGG Pathways	Terms/Pathway ID
1	16767735	*ESR1*	397435	Gene expression and signaling by G protein-coupled receptors (GPCR).	GO:0007200
4	51508361	*RIPK2*	100518835	Signaling by GPCR and immune system.	ssc04621, ssc04722
7	37570312	*FGD2*	100524814	Signaling by GPCR and signaling by Rho GTPases.	GO:0035023, GO:0007200
9	43255454	*FDX1*	397133	Related pathways are metabolism and infectious disease	IPR012675
13	29861003	*ABHD5*	497624	Lipoprotein metabolism and metabolism.	GO:0019915,GO:0051006
13	34681199	*PRKAR2A*	397493	Platelet activation, signaling and aggregation and sertoli–sertoli cell junction dynamics	GO:1903538,GO:1904146
13	34775057	*PFKFB4*	100158056	Metabolism and glycosaminoglycan metabolism.	GO:0003873
13	35107691	*USP4*	100512072	Signaling by GPCR and Tumor necrosis factor (TNF) signaling	GO:0007200, GO:0006511
14	30401893	*NCOR2*	733643	gene expression and signaling by GPCR.	GO:0007200

^1^ Derived from Sus scrofa Build 10.2.

## Supplementary Materials

The following are available online at https://www.mdpi.com/2076-2615/9/6/314/s1, Table S1: The estimated average observed heterozygosity (*He*), Table S2A: The inbreeding coefficient of the inbreeding and non-inbreeding pigs, Table S2B: Correlation inbreeding coefficient F and F(ROH) across generations in the inbreeding and non-inbreeding pigs, Table S2C: Student t-tests between inbreeding coefficients of the inbreeding and non-inbreeding pigs, Table S3: ROH region at inbred and non-inbred strain for each generation.
